# Soil pathogens shift root strategies from acquisition to defense under shaded understorey conditions in *Pinus koraiensis* seedlings

**DOI:** 10.48130/forres-0026-0024

**Published:** 2026-07-22

**Authors:** Lulu Xie, Xiaohan Li, Xiulong Zhang, Jinguo Wang, Dapao Yu, Qing-Wei Wang

**Affiliations:** 1 CAS Key Laboratory of Forest Ecology and Silviculture, Institute of Applied Ecology, Chinese Academy of Sciences, Shenyang, Liaoning 110016, China; 2 Ministry of Education Key Laboratory of Ecology and Resource Use of the Mongolian Plateau, Inner Mongolia Key Laboratory of Grassland Ecology, and School of Ecology and Environment, Inner Mongolia University, Huhhot, Inner Mongolia Autonomous Region 010021, China; 3 Changbaishan National Field Observation and Research Station for Forest Ecosystem, Yanbian Korean Autonomous Prefecture, Jilin 133613, China; 4 Academy of Forestry, Inner Mongolia Agricultural University, Hohhot, Inner Mongolia Autonomous Region 010021, China

**Keywords:** Biomass allocation, Seedling survival, Specific root length, Trade-off, Root economics spectrum

## Abstract

Broadleaved Korean pine forest, a typical zonal vegetation type in Northeastern China, is important in maintaining regional ecological security. However, the regeneration of Korean pine (*Pinus koraiensis*) seedlings remains a major constraint in the primary forests. Although declining light availability and increasing soil pathogens are recognized as critical factors affecting seedling survival and growth, their interactive effects and underlying mechanisms remain unclear. In this study, we conducted a controlled experiment with three light levels (200, 400, and 600 μmol·m^−2^·s^−1^) and four soil pathogen concentrations (0, 1 × 10^4^, 1 × 10^6^, and 1 × 10^8^ CFU/mL) to examine seedling survival, growth, physiology, and above- and belowground functional traits. Low light significantly reduced survival, relative growth rate, photosynthesis, and biomass of *P. koraiensis* seedlings, while the presence of high pathogen concentrations exacerbated these impacts, notably driving mortality rates up by 95%. Leaf traits were only regulated by light, with shaded conditions increasing specific leaf area and nitrogen (N) concentration, but decreasing total phenolics and carbon/nitrogen ratio. In contrast, root traits were jointly influenced by light and soil pathogens. Under low light, seedlings exhibited an acquisitive strategy characterized by higher specific root length and N concentration, along with reduced root diameter and defense investment. However, increasing pathogen concentration reversed this pattern, suppressing resource acquisition traits while enhancing root diameter and defensive compounds (total phenolics and tannins). These results indicate that light determines the baseline resource context, while soil pathogens impose additional constraints that shift root strategies from resource acquisition to defense. Such interactive regulation modulates seedling establishment, with critical implications for improving forest regeneration and ecosystem stability.

## Introduction

Forest natural regeneration is a key process underpinning ecosystem recovery and the maintenance of forest functions, driving the renewal of forest resources and sustaining ecosystem services^[1−3]^. The Northeast China forest region is a significant forest belt within China's ecological security framework and plays a key role in supporting national development^[4,5]^. Primary broadleaved mixed Korean pine forest, as the zonal climax vegetation of this region, is essential for maintaining ecological security in Northeast Asia and mitigating climate change^[6,7]^. However, the dominant species, Korean pine (*Pinus koraiensis* Siebold & Zucc.), exhibits poor regeneration, which constrains the functioning and stability of these forests, most of which have been replaced by secondary forests^[8−10]^. Therefore, the exploration of the mechanisms underlying *P. koraiensis* regeneration is urgently required.

Light availability acts as a primary driver of plant photosynthesis and regulates key physiological processes, including growth, development, and carbon (C) balance^[11−13]^. It is widely recognized as a critical factor controlling *P. koraiensis* regeneration^[6,14,15]^. Previous studies have demonstrated that *P. koraiensis* regeneration is generally successful in secondary forests but limited in primary forests, largely due to poor light availability, which restricts seedling performance^[5,8,14]^. However, these conclusions are largely based on light-centered perspectives, and the mechanisms remain incompletely resolved. Recent evidence highlights soil-borne pathogens as an additional key factor influencing understorey seedling establishment and dynamics^[16,17]^. Along the succession from secondary to primary forests, increasing tree diversity and litter inputs promote the accumulation of soil pathogens, which can directly damage roots and increase seedling mortality^[18,19]^, and may interact with light availability to further constrain growth under low-light conditions^[20]^. However, the interactive effects of aboveground (light availability) and belowground (soil pathogens) factors have received limited attention, hindering a comprehensive understanding of regeneration processes.

Plant functional traits provide an effective framework for linking plant performance to environmental conditions and adaptive strategies^[21,22]^. The plant economics spectrum, including leaf economics spectrum (LES) and root economics spectrum (RES), describes trade-offs between resource acquisition and conservation^[23,24]^. The LES reflects variation in growth–survival strategies; for example, specific leaf area (SLA) and nitrogen (N) concentration are associated with resource acquisition capacity, with high SLA and leaf N indicating a fast-growth strategy^[25,26]^, whereas leaf phenolic contents correspond to enhanced stress resistance^[12,27]^. Similarly, the RES characterizes belowground strategies, where acquisitive species typically exhibit high root N concentration and specific root length (SRL), while conservative species develop thicker roots, lower N, and higher tissue density (RTD)^[28−30]^. Importantly, plant traits are coordinated across organs, and their trade-offs enable adaptation to complex environmental conditions^[24,31,32]^. Light primarily affects leaf traits, whereas soil pathogens directly influence root traits and function. However, how *P. koraiensis* seedlings coordinate these responses under simultaneous variation in light and soil pathogens remains unclear. According to optimal partitioning theory, plants tend to allocate biomass to organs that capture the most limiting resource, thereby maximizing growth and fitness^[33,34]^. Therefore, investigating the allocation patterns and plant trait adjustments is critical for understanding regeneration strategies.

In this study, we investigated how *P. koraiensis* seedlings responded to variations in light availability and soil pathogens. An indoor experiment was conducted to assess their effects on seedling growth, survival, and photosynthetic performance. Through a comprehensive analysis of above- and belowground functional traits, we aimed to elucidate the adaptive strategies of *P. koraiensis* seedlings and provide a deeper understanding of the underlying mechanisms of forest regeneration. The following hypotheses were proposed: (1) Consistent with optimal partitioning theory^[33]^, low light limits carbon gain, forcing plants to prioritize aboveground growth to maximize light capture^[2]^. We hypothesize that the infection of soil pathogens will impose a secondary metabolic cost—diverting carbon away from already constrained growth to support defense—thereby causing a synergistic reduction in seedling performance. (2) *P. koraiensis* seedlings will shift toward an acquisitive leaf strategy (e.g., increased specific leaf area) to maximize light interception under low-light conditions. However, we expect that high pathogen concentration will truncate this adaptive response, as the plant is forced to reallocate resources from leaf production toward resistance^[32]^, thus effectively constraining its ability to pursue a purely acquisitive strategy. (3) We then expect that seedlings under low light will shift biomass toward aboveground tissues to optimize light acquisition at the expense of root defense according to the growth–defense trade-off framework. Consequently, we hypothesize that the presence of pathogens will force a strategy redirection, where the plant will abandon the optimized light-acquisition model to bolster root defense, leading to a breakdown in the expected coordination between aboveground growth and root development.

## Materials and methods

### Experimental design

The experiment was conducted at the Shenbei laboratory of the Institute of Applied Ecology, Chinese Academy of Sciences, Shenyang, China (41.90° N, 123.60° E). Five growth chambers were used, each employing a factorial design comprising three light levels and four soil pathogen concentrations, resulting in 12 treatments. Each growth chamber contained three shelf tiers for three distinct light treatments, with every tier wrapped in black shade cloth to prevent cross-light interference. Light levels were set at 200, 400, and 600 μmol·m^−2^·s^−1^, representing low (L1), medium (L2), and high (L3) light conditions, respectively, which were controlled using LED sources and measured with a Li-1500 irradiance meter (Li-COR, USA). Light levels were based on field measurements of understorey light availability across successional stages in mixed broadleaf–Korean pine forests^[6]^. Soil pathogen treatments included four concentrations of *Fusarium oxysporum* spore suspension: 0, 1 × 10^4^, 1 × 10^6^, and 1 × 10^8^ CFU/mL, representing the control, low, medium, and high pathogen conditions, respectively. The *F. oxysporum* strain was isolated from the roots of *P. koraiensis* seedlings and identified as the dominant pathogen taxon through morphological and molecular analyses^[35]^. Previous research has verified that inoculation with the high-concentration *F. oxysporum* strain (1 × 10^8^ CFU/mL) can induce typical pathogenic symptoms in host plants, including root rot and white mycelium coverage on root surfaces^[35]^ (Supplementary Fig. S1). The *F. oxysporum* strain was obtained from the Microbial Culture Collection of the College of Forestry, Northeast Forestry University. This gradient of *F. oxysporum* concentration was designed based on the pre-experimental survey of its abundance in forest soils, which can effectively simulate the gradient variation of soil pathogen concentration under natural forest understorey environments. We acknowledge that natural forest soils host a complex microbial community, and this design may not fully capture the diversity of soil pathogens encountered in field conditions. However, the chosen concentrations provide insight into the potential impact of soil pathogens on seedling performance.

In March 2024, well-filled seeds of *P. koraiensis* were surface-sterilized with 0.3% K_2_MnO_4_ for 15 min, rinsed with sterile distilled water, and germinated on 0.8% agar plates at 24 °C. Germinated seeds were then transplanted into sterilized pots containing a mixed substrate and grown in climate-controlled chambers under a 16-h photoperiod, a temperature of 23–25 °C, and relative humidity of 60%. The soil substrate was collected from the topsoil layer (0–10 cm) of a broadleaf–Korean pine forest, sieved through a 1 mm mesh, and mixed with sand at a ratio of 3:1 (v/v). The mixture was sterilized using gamma irradiation (> 25 kGy). One week after transplanting, uniform seedlings were inoculated with *F. oxysporum*. The fungus was cultured on PDA medium for 5 d at 26 °C, and transferred to liquid PD medium and incubated at 28 °C with shaking (160 rpm) for 7 d. Spore concentration was determined using a spectrophotometer (OD600)^[35]^, yielding approximately 1.0 × 10^8^ CFU/mL, and subsequently diluted to 1 × 10^6^ and 1 × 10^4^ CFU/mL. Each pot received 50 mL of the corresponding fungal suspension via syringe injection, while control pots received an equal volume of sterile water. Each treatment included 20 seedlings. After inoculation, seedlings were maintained under the designated light conditions. Pots were rotated weekly to minimize positional effects. A commercial fertilizer (N : P : K = 6%:10%:6%, HYPONEX, Japan) was applied every 2 weeks to ensure sufficient nutrient supply.

### Sampling and processing

Seedling height and basal diameter were measured periodically throughout the experiment to calculate relative growth rates. In August 2024, final sampling was conducted, and survival rates were recorded. Firstly, five seedlings per treatment per chamber were randomly selected for gas exchange measurements. After this, seedlings were carefully harvested, rinsed with sterile water, and separated into leaves, stems, and coarse roots (diameter > 2 mm) and fine roots (diameter ≤ 2 mm). Samples were transported to the laboratory and stored at 4 °C for further analyses, including measurements of functional traits and biomass.

### Measurements and analytical methods

Gas exchange parameters were measured between 09:00 and 11:00 using a LI-6400 portable photosynthesis system (Li-Cor, USA)^[36]^. A red–blue light source (1,000 μmol·m^−2^·s^−1^ photosynthetically active radiation) was used in the leaf chamber. CO_2_ concentration was maintained at approximately 400 μmol·mol^−1^ using a buffer system. The following parameters were obtained: net photosynthetic rate (*P*_n_), stomatal conductance (*G*_s_), transpiration rate (*T*_r_), and intercellular CO_2_ concentration (*C*_i_). Relative growth rate (RGR) was calculated as: RGR = [ln (D_b_^2^ × H_b_) – ln (D_a_^2^ × H_a_)]/(T_b_ − T_a_), where D is basal diameter, H is seedling height, and T represents time at measurements a and b^[12]^.

Fresh leaves were scanned using a CanoScan scanner, and leaf area was determined using ImageJ software. SLA (cm^2^ g^−1^) was calculated as leaf area divided by leaf mass. Fine roots were classified into absorptive roots (1^st^–2^nd^ order) and transport roots, and all root trait analyses were performed on absorptive roots. Here, roots were scanned using an EPSON Perfection V800 scanner and analyzed with WinRHIZO software (Regent Instruments, Canada) to obtain root diameter, length, surface area, and volume. After scanning, absorptive roots were oven-dried at 65 °C to constant weight. Specific root length (SRL, m g^−1^) was calculated as root length per unit dry mass, and root tissue density (RTD, g cm^−3^) was calculated as dry mass divided by root volume^[37]^. Plant tissues (roots, stems, and leaves) were heated at 105 °C for 30 min and dried at 65 °C to a constant weight. The root-to-shoot ratio (R/S) was calculated as root biomass divided by the sum of stem and leaf biomass. Dried samples were ground to pass through an 80-mesh sieve. C and N concentrations were determined using an elemental analyzer (Elementar III, Germany). Total phenolics and condensed tannins were measured using the Folin–Ciocalteu method^[2]^.

### Data analysis

In each chamber, the five seedlings of each treatment were considered as one independent biological replicate. Linear mixed-effect models were used to explore the effects of light, soil pathogens, and their interactions on seedling growth, physiology, and functional traits, with chamber as a random factor, using the *nlme* package^[12]^. When significant effects were detected, post-hoc multiple comparisons were performed using Tukey's test (*p* < 0.05), and *p*-values were corrected with the Benjamini–Hochberg (BH) method^[38]^. Principal component analysis (PCA) was conducted to identify major axes of variation in leaves and fine-root traits. All analyses were performed with R 4.0.3 (R Development Core Team, 2023)^[39]^.

## Results

### Growth and photosynthetic traits of *Pinus koraiensis* seedlings

**Figure 1:**
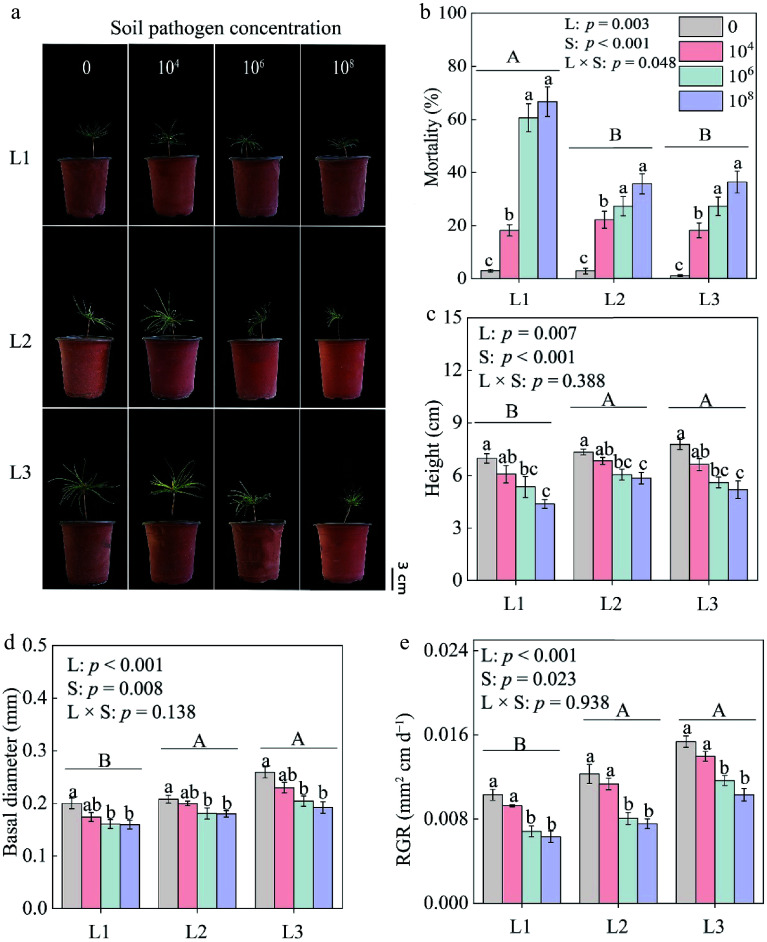
Growth response of *P. koraiensis* seedlings to different light and soil pathogen treatments: (a) phenotype, (b) mortality, (c) height, (d) basal diameter, and (e) relative growth rate (RGR). Error bars show the mean values and SE (*n* = 5). L: light intensity; S: soil pathogen; L × S: the interaction of light intensity and soil pathogen; L1, L2, L3: low, medium, and high light at 200, 400, and 600 μmol·m^−2^·s^−1^, respectively; 0, 1 × 10^4^, 1 × 10^6^, 1 × 10^8^: the concentration of *Fusarium oxysporum* at 0, 1 × 10^4^, 1 × 10^6^, 1 × 10^8^ CFU/mL, respectively. Different uppercase letters indicate significant differences among light treatments; lowercase letters indicate significant differences among pathogen concentrations under the same light level (*p* < 0.05).

Compared with high light, *P. koraiensis* seedlings treated with low light exhibited poorer growth performance, a difference that was particularly pronounced when soil pathogen concentrations increased (Fig. 1a). Seedling mortality under low light was significantly higher than that under medium and high light, and it gradually increased with rising pathogen concentration, peaking at 1 × 10^8^ CFU/mL (Fig. 1b). Furthermore, light and soil pathogens significantly affected the growth of *P. koraiensis* seedlings. The results showed that low light significantly reduced seedling height, basal diameter, and RGR (*p* < 0.05), and these parameters were further suppressed as pathogen concentration increased (Fig. 1c–e).

Plant aboveground, belowground, and total biomass were significantly affected by light, soil pathogens, and their interaction (*p* < 0.001) (Table 1). Overall, plant biomass exhibited a trend of low light < medium light < high light, and the values in the control were significantly higher than those in the 1 × 10^8^ CFU/mL treatment. The root-to-shoot ratio (R/S) increased with light and pathogen concentration, but remained below 1 across treatments, indicating that more biomass was allocated to the aboveground parts. Light and soil pathogens also significantly affected physiological traits. Under low light, net photosynthetic rate (*P*_n_) and transpiration rate (*T*_r_) were significantly lower than under high light (*p* < 0.001). Across all light treatments, *P*_n_ under the control was significantly higher than that under high pathogen concentration (*p* < 0.05) (Table 1).

**Table 1:** Photosynthetic traits and biomass allocation of *P. koraiensis* seedlings across light and soil pathogen treatments.

Light level	Pathogen concentration (CFU/mL)	Aboveground biomass (g)	Belowground biomass (g)	Total biomass (g)	Root : shoot ratio	*P*_n_ (μmol·m^–2^·s^–1^)	*T*_r_ (mmol·m^-2^·s^−1^)
L1	0	0.23 ± 0.01Ca	0.11 ± 0.01Ca	0.34 ± 0.02Ca	0.49 ± 0.02Bb	3.56 ± 0.44Ca	0.36 ± 0.05Ba
	1 × 10^4^	0.15 ± 0.01Cb	0.07 ± 0.01Cb	0.23 ± 0.01Cb	0.51 ± 0.04Bab	2.69 ± 0.16Cb	0.36 ± 0.05Ba
	1 × 10^6^	0.13 ± 0.01Cbc	0.07 ± 0.01Cbc	0.20 ± 0.01Cbc	0.54 ± 0.01Bab	2.35 ± 0.09Cbc	0.34 ± 0.03Ba
	1 × 10^8^	0.11 ± 0.01Cc	0.06 ± 0.01Cc	0.17 ± 0.01Cc	0.56 ± 0.07Ba	1.75 ± 0.26Cc	0.34 ± 0.02Ba
L2	0	0.32 ± 0.01Ba	0.18 ± 0.01Ba	0.49 ± 0.02Ba	0.51 ± 0.02Bb	4.20 ± 0.19Ba	0.40 ± 0.01Ba
	1 × 10^4^	0.26 ± 0.01Bb	0.13 ± 0.01Bbc	0.39 ± 0.03Bb	0.57 ± 0.02Bab	3.51 ± 0.32Bab	0.27 ± 0.03Bb
	1 × 10^6^	0.21 ± 0.02Bc	0.12 ± 0.01Bbc	0.33 ± 0.02Bc	0.59 ± 0.04Bab	3.36 ± 0.38Bab	0.26 ± 0.04Bb
	1 × 10^8^	0.16 ± 0.01Bd	0.09 ± 0.01Bc	0.26 ± 0.01Bd	0.64 ± 0.05Ba	3.07 ± 0.05Bb	0.22 ± 0.03Bb
L3	0	0.47 ± 0.01Aa	0.27 ± 0.02Aa	0.74 ± 0.01Aa	0.59 ± 0.04Ab	7.06 ± 0.03Aa	0.61 ± 0.02Aa
	1 × 10^4^	0.33 ± 0.01Ab	0.21 ± 0.01Ab	0.53 ± 0.01Ab	0.63 ± 0.04Aab	5.53 ± 0.46Ab	0.57 ± 0.05Aa
	1 × 10^6^	0.29 ± 0.02Ac	0.17 ± 0.01Ab	0.46 ± 0.03Ac	0.62 ± 0.07Aab	4.01 ± 0.28Ac	0.43 ± 0.04Ab
	1 × 10^8^	0.17 ± 0.02Ad	0.13 ± 0.01Ac	0.30 ± 0.02Ad	0.75 ± 0.06Aa	3.56 ± 0.11Ac	0.29 ± 0.01Ac
L	F	151.78	165.93	254.54	6.87	82.16	26.80
	*P*	< 0.001	< 0.001	< 0.001	0.003	< 0.001	< 0.001
S	F	116.76	57.16	144.78	3.11	34.12	11.36
	*P*	< 0.001	< 0.001	<0.001	0.038	< 0.001	< 0.001
L × S	F	9.41	5.11	11.81	0.55	5.21	4.08
	*P*	< 0.001	< 0.001	< 0.001	0.769	< 0.001	0.003
Different uppercase letters indicate significant differences among light treatments, and lowercase letters indicate significant differences among pathogen concentrations under the same light (*p* < 0.05). L: light intensity; S: soil pathogens; L × S: interaction effect. L1, L2, L3: low, medium, and high light at 200, 400, and 600 μmol·m^−2^·s^−1^, respectively; 0, 1 × 10^4^, 1 × 10^6^, 1 × 10^8^: the concentration of *Fusarium oxysporum* at 0, 1 × 10^4^, 1 × 10^6^, 1 × 10^8^ CFU/mL, respectively.							

### Plant functional traits of *Pinus koraiensis* seedlings

**Figure 2:**
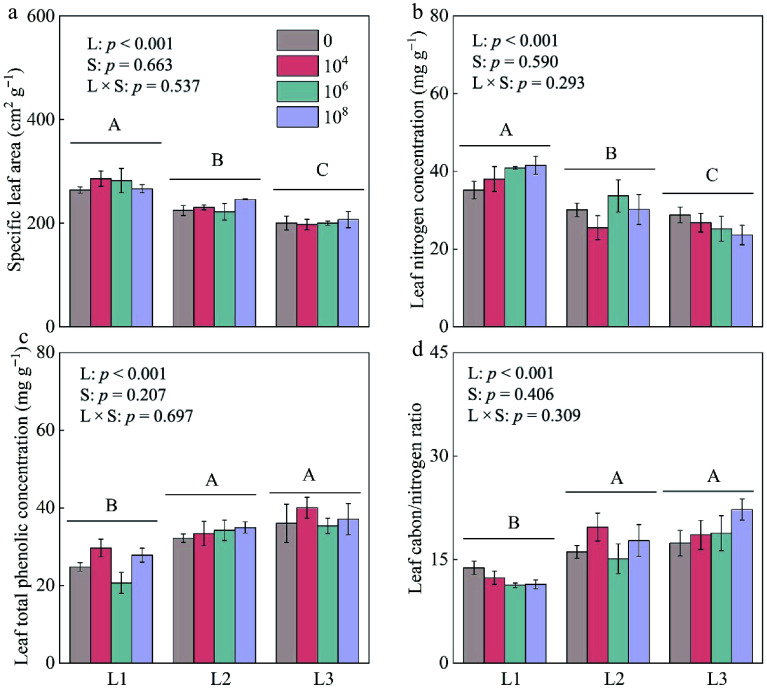
Variation in leaf traits of *P. koraiensis* seedlings across light and soil pathogen treatments: (a) specific leaf area; (b) N concentration; (c) total phenolic concentration; and (d) leaf carbon-to-nitrogen ratio. Error bars show the mean values and SE (*n* = 5). L: light intensity; S: soil pathogen; L × S: the interaction of light intensity and soil pathogen. L1, L2, L3: low, medium, and high light at 200, 400, and 600 μmol·m^−2^·s^−1^, respectively; 0, 1 × 10^4^, 1 × 10^6^, 1 × 10^8^: the concentration of *Fusarium oxysporum* at 0, 1 × 10^4^, 1 × 10^6^, and 1 × 10^8^ CFU/mL, respectively. Different uppercase letters indicate significant differences among light treatments, and lowercase letters indicate significant differences among pathogen concentrations under the same light level (*p* < 0.05).

Light had a significant effect on leaf traits (*p* < 0.05), whereas the effect of soil pathogens was not significant (Fig. 2). Low light significantly promoted acquisitive traits, such as SLA and N concentration (Fig. 2a, b), but suppressed conservative traits, including total phenolic concentration and C/N ratio (Fig. 2c, d).

**Figure 3:**
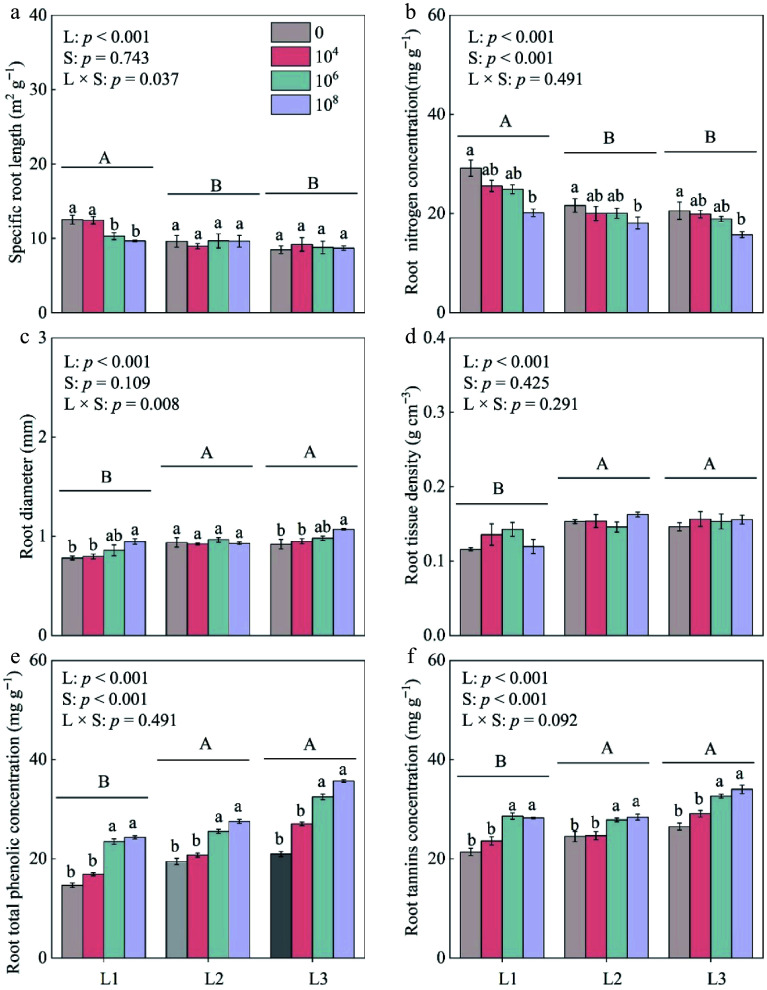
Variation in root functional traits of *P. koraiensis* seedlings across light and soil pathogen treatments: (a) specific root length; (b) N concentration; (c) diameter; (d) root tissue density; (e) total phenolic concentration; and (f) tannin concentration. Error bars show the mean values and SE (*n* = 5). L: light intensity; S: soil pathogen; L × S: the interaction of light intensity and soil pathogen. L1, L2, L3: low, medium, and high light at 200, 400, and 600 μmol·m^−2^·s^−1^, respectively; 0, 1 × 10^4^, 1 × 10^6^, 1 × 10^8^: the concentration of *Fusarium oxysporum* at 0, 1 × 10^4^, 1 × 10^6^, and 1 × 10^8^ CFU/mL, respectively. Different uppercase letters indicate significant differences among light treatments, and lowercase letters indicate significant differences among pathogen concentrations under the same light level (*p* < 0.05).

The interaction between light and soil pathogens significantly affected SRL (*p* = 0.037). SRL under low light was significantly higher than that under high light, and reached its minimum in the 1 × 10^8^ CFU/mL treatment. However, under medium and high light, differences among different pathogen concentrations were not significant (Fig. 3a). Root N concentration was significantly higher under low light than under other light treatments (*p* < 0.001), and was inhibited by high pathogen concentration across all light treatments (Fig. 3b). Low light significantly reduced root diameter, while root diameter increased from the control to high pathogen concentration under low and high light (Fig. 3c). RTD increased with light availability (*p* < 0.001), with significantly lower values under low light than under medium and high light (Fig. 3d). Root total phenolics and tannin concentrations varied significantly with light (*p* < 0.001), both reaching minimum values under low light, while higher pathogen concentrations significantly increased both compared to control (*p* < 0.001) (Fig. 3e, f).

### Principal component analysis of functional traits

**Figure 4:**
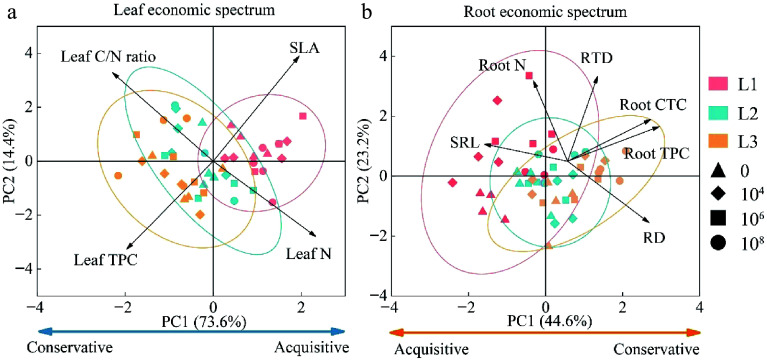
Principal component analysis (PCA) of leaf and root traits of *P. koraiensis* seedlings across light and soil pathogen treatments. SLA, specific leaf area; leaf N, leaf nitrogen concentration; leaf TPC, leaf total phenolic concentration; leaf C/N ratio, leaf carbon/nitrogen ratio; SRL, specific root length; root N, root nitrogen concentration; RD, root diameter; RTD, root tissue density; root TPC, root total phenolic concentration; root CTC, root tannin concentration. L1, L2, L3: low, medium, and high light at 200, 400, and 600 μmol·m^−2^·s^−1^, respectively; 0, 1 × 10^4^, 1 × 10^6^, 1 × 10^8^: the concentration of *Fusarium oxysporum* at 0, 1 × 10^4^, 1 × 10^6^, and 1 × 10^8^ CFU/mL, respectively.

For leaf traits, principal component axis 1 (PC1) and axis 2 (PC2) explained 73.6% and 14.4% of the total variance, respectively. Leaf traits associated with resource acquisition (SLA and leaf N concentration) were positioned on the positive side of PC1 and dominated under low light. In contrast, seedlings tended to adopt conservative strategies (higher total phenolics and C/N ratio) under high light (Fig. 4a). With regard to root traits, the first two PCA axes together explained 67.8% of the total trait variation. PC1 represented the trade-off along the root economics spectrum between acquisition and defense strategies (Fig. 4b). Under high light, defensive traits such as root total phenolics and tannins were located on the positive side of PC1. In contrast, low light was associated with acquisitive traits (higher SRL and root N concentration), and this tendency was attenuated along soil pathogen concentration (Fig. 4b). Samples under medium light were consistently located near the center of the PCA space, indicating no clear adaptive strategy (Fig. 4b).

## Discussion

**Figure 5:**
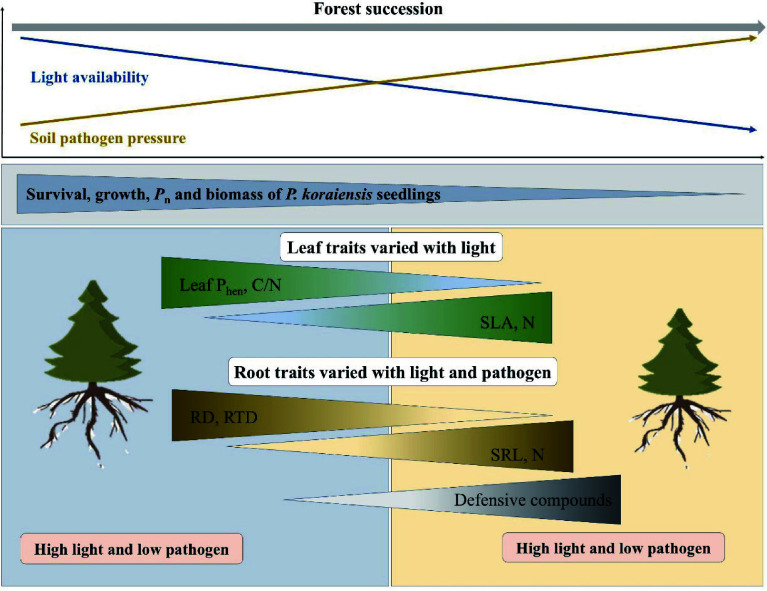
The conceptual model illustrating adaptation strategies of *P. koraiensis* seedlings to variation in light availability and soil pathogens. SLA, specific leaf area; N, nitrogen concentration; leaf P_hen_: leaf total phenols concentration; SRL, specific root length; C/N ratio, carbon/nitrogen ratio; SRL, specific root length; RD, root diameter; RTD, root tissue density; defensive compounds, including root total phenols and tannin concentration.

In this study, we explored variation in survival, growth, and physiology of *P. koraiensis* seedlings, as well as their adaptive strategies in both leaves and roots in response to light availability and *F. oxysporum* (a representative soil-borne pathogen) infection (Fig. 5). As expected, low light significantly inhibited the growth and survival of *P. koraiensis* seedlings, and this negative effect was further exacerbated by increasing pathogen concentration. Seedlings under low light adopted an acquisitive strategy in both leaves and roots, while the pathogen only modified root traits. High pathogen concentration under low light increased root diameter and defensive compounds, indicating a shift toward structural reinforcement and chemical defense. This trade-off reflects a strategic reallocation of resources from acquisition to defense, highlighting root plasticity in responding to biotic stress under light limitation. Together, these findings suggest that the coordination between light limitation and soil pathogens plays a critical role in shaping *P. koraiensis* regeneration, which provides important implications for future forest management and restoration strategies.

### Soil pathogens exacerbated the inhibition of low light on the survival and growth of *Pinus koraiensis* seedlings

It is widely accepted that the regeneration of *P. koraiensis* is largely dependent on understorey light conditions, with seedlings exhibiting faster growth under high light^[5,9,15]^. Our results support this pattern: survival and RGR of *P. koraiensis* seedlings under low light were significantly lower than under high light (Fig. 1). This suggests that reduced canopy openness in primary forests is unfavorable for the growth and regeneration of *P. koraiensis* seedlings^[6,8]^. Along the succession of broadleaf–Korean pine forests, the dense canopy of primary forests intercepts most of the photosynthetically active radiation, resulting in an understorey light transmittance of only approximately 2.2%^[40]^. Compounding this limitation, light reaching the forest floor is predominated by diffuse radiation, which further constrains seedling carbon gain and growth^[41]^. In addition, the accumulation of litter layers and increased species diversity along succession promote the proliferation of soil-borne pathogens^[41,42]^. A recent study has reported that soil pathogens lead to high mortality rates in *P. koraiensis* seedlings^[35]^. Consistent with these findings, our results reveal that increasing soil pathogen concentration significantly increased seedling mortality and reduced RGR and biomass (Fig. 1; Table 1). This is primarily because seedling roots are highly susceptible to pathogen infection, leading to root rot or wilting, or indirectly impairing water and nutrient uptake, thereby restricting growth^[16,43,44]^.

According to the functional equilibrium theory, plants allocate more biomass to the organs responsible for acquiring the most limiting resources^[33,34]^. In this study, *P. koraiensis* seedlings exhibited a lower R/S under low light, indicating greater allocation to aboveground organs to maximize light capture^[45−47]^. However, as pathogen concentration increased, R/S increased under low light, reflected by a shift toward greater investment in roots to enhance defense capacity. This interpretation is supported by the increased concentrations of root total phenolics and tannins (Fig. 3), which are important defensive compounds against pathogens^[48,49]^. Importantly, our findings further suggest that the key determinant of seedling growth and survival lies in the interaction between light and soil pathogens, rather than any single factor. Low light significantly limited survival, growth, and biomass accumulation, while soil pathogens exacerbated these negative effects (Table 1). This finding complements the traditional 'light limitation hypothesis', indicating that regeneration failure in mixed broadleaf–Korean pine forests may arise from the interactive regulation of understory light conditions and soil pathogen stress, rather than light limitation alone.

### Adaptation strategy of roots shifts from acquisition to conservation with increasing soil pathogen concentration under low light

Our results indicate that light is the primary driver of functional trait variation in *P. koraiensis* seedlings, prompting a marked shift in their resource-use strategies along the acquisition–defense conservation spectrum (Fig. 4). By contrast, soil pathogens modulate this strategy through root restructuring and the accumulation of defensive compounds, with significant interaction effects between light and pathogen on root morphology and nutrient-related traits. Specifically, under low light, seedlings exhibited a typical resource-acquisitive strategy, manifested by higher SLA, SRL, and N concentrations (Fig. 4). Such trait combinations can enhance light capture and nutrient uptake efficiency^[24,32,50]^, allowing seedlings to cope with resource-limited conditions. This pattern aligns with a theory predicting that plants in shaded environments tend to adopt acquisitive traits to maximize photosynthetic returns^[25,51]^. Conversely, under high light, seedlings shifted toward a more conservative strategy, reflected by increased RTD, C/N ratios, and greater defensive metabolites (such as total phenolics and condensed tannins). These results are in line with the growth–defense trade-off hypothesis^[52]^, which predicts that C surplus under favorable conditions is preferentially invested in secondary metabolism to strengthen defense.

In addition, the findings revealed that soil pathogens mainly affected root traits, not leaf traits, suggesting that the leaf-trait hypothesis was not fully supported. In this present study, root traits, rather than leaf traits, were significantly reshaped by interactions between soil pathogens and light (Fig. 3). Under low light, increasing pathogen concentration reduced SRL and increased root diameter, indicating a shift in root construction strategy^[22,23]^. This finding highlights the critical role of biotic stress in shaping root morphology. In this regard, seedlings tended to reallocate limited C resources toward defense-related structures, even under low-light conditions (Fig. 5). Root thickening may act as a physical barrier against pathogen invasion^[53,54]^, but this occurs at the expense of root elongation and exploratory capacity. Such trait shifts reflect a trade-off in roots between resource acquisition and defense investment, consistent with previous findings that plants adjust root traits to balance nutrient uptake and defense requirements^[55−58]^. Collectively, our findings illustrate that *P. koraiensis* seedlings coordinate root traits to adapt to combined aboveground (light availability) and belowground (*F. oxysporum* infection) stresses. This interaction ultimately governs the balance between growth and survival, highlighting a key ecological mechanism underlying regeneration limitation in the forest understorey.

### Implications of growth regulation mechanisms for forest conservation

Overall, our results indicate that forest seedlings may have evolved flexible ecological strategies adapting to the combined effects of aboveground abiotic and belowground biotic factors. On the basis of the traditional light limitation hypothesis^[5,6,15]^, the present study highlights that soil pathogens (here using *F. oxysporum* as a typical representative soil-borne pathogen), interacting with light, represent a critical but often overlooked constraint during seedling establishment. This interaction suggests that regeneration limitation cannot be explained by single-factor hypotheses, but rather arises from the imbalance between light availability and pathogen stress. Our findings not only advance our understanding of regeneration mechanisms, but also provide a mechanistic basis for forest conservation practices. Several management implications can be proposed. First, the implementation of controlled, selective thinning is critical. Based on our findings that light limitation significantly hampers performance, we recommend a moderate thinning intensity. This practice should aim to increase understory light transmittance. This appropriate light level will optimize photosynthetic capacity while preventing excessive forest understorey disturbance, thereby minimizing the risk of rapid pathogen proliferation associated with sudden environmental shifts. It is worth emphasizing that this parameter is summarized from controlled chamber experiments and cannot be directly generalized to heterogeneous natural forest ecosystems without cross-site field verification. Second, we advocate for the targeted regulation of soil pathogens via rational mixed-species configuration. Rather than relying solely on general microbial amendments, forest managers should consider interplanting *P. koraiensis* with specific tree or shrub species that act as natural suppressors of common soil pathogens. By strategically designing forest structures that enhance plant-derived chemical or biological antagonism in the rhizosphere, managers can effectively reduce root infection risks, enabling seedlings to shift their internal resources from defensive investments back toward growth-oriented strategies.

Additionally, despite providing new insights into the interactive effects of light and soil pathogens on *P. koraiensis* regeneration, several limitations should be acknowledged. This study was conducted under controlled experimental conditions, which inevitably simplified the complexity of natural forest ecosystems. Soil pathogens were applied as *F. oxysporum* concentration gradients rather than representing the full diversity and dynamics of natural soil biota. Besides, the experiment focused on early seedling stages and short-term responses. Longer-term processes, including ontogenetic shifts and acclimation, were not captured but may play a critical role in determining regeneration success. Furthermore, while this controlled experiment provides evidence that light limitation and pathogen inoculation jointly affect seedling performance and root traits, these findings represent mechanistic insights under standardized conditions. Therefore, caution should be exercised when extrapolating this experiment to conclude that this interaction is the primary driver of natural regeneration failure in complex field environments. Last, the analysis of survival data should be better justified in further studies, because binomial mortality data may require a statistical model different from that used for continuous growth and trait variables. We thus note that future experiments should include repeated monitoring of seedling survival through time, which would allow the application of more appropriate survival or binomial-response models and provide a more rigorous assessment of mortality dynamics. In summary, while our controlled experimental approach allowed us to disentangle the independent and interactive effects of light availability and representative soil-borne pathogen stress on seedling performance, field-based *in situ* manipulation experiments and multi-scale integration with controlled laboratory studies are urgently needed to validate these uncovered mechanisms under realistic heterogeneous environmental conditions, and further improve the field ecological applicability of our obtained findings.

## Conclusions

This study elucidates the core mechanistic basis of *Pinus koraiensis* regeneration via controlled experiments, revealing that the interactive effect of light availability and soil pathogens could provide mechanistic evidence for seedling establishment in forest understorey. Mechanistically, light constructs the fundamental resource acquisition framework and constrains seedling carbon allocation patterns, while soil pathogens serve as a critical selective stress that redirects carbon investment from growth to defense. The trade-off between light-driven growth potential and pathogen-induced defense requirements ultimately determines seedling establishment. This mechanistic insight into the coupled regulation of abiotic light conditions and biotic soil pathogen stress provides a targeted theoretical basis for optimizing *P. koraiensis* regeneration management and sustaining temperate forest ecosystem stability.

## SUPPLEMENTARY DATA

Supplementary data to this article can be found online.

## Data Availability

The datasets generated during and/or analyzed during the current study are available from the corresponding author on reasonable request.
