# Supplementary material for: Soil pathogens shift root strategies from acquisition to defense under shaded understorey conditions in Pinus koraiensis seedlings
Source: For Res (Fayettev). 2026 Jul 22;6:e023. doi: 10.48130/forres-0026-0024 (PMC13447282; doi:10.48130/forres-0026-0024)
Supplement: Supplementary file 1 [file FR-2026-6-0024-S1.pdf]

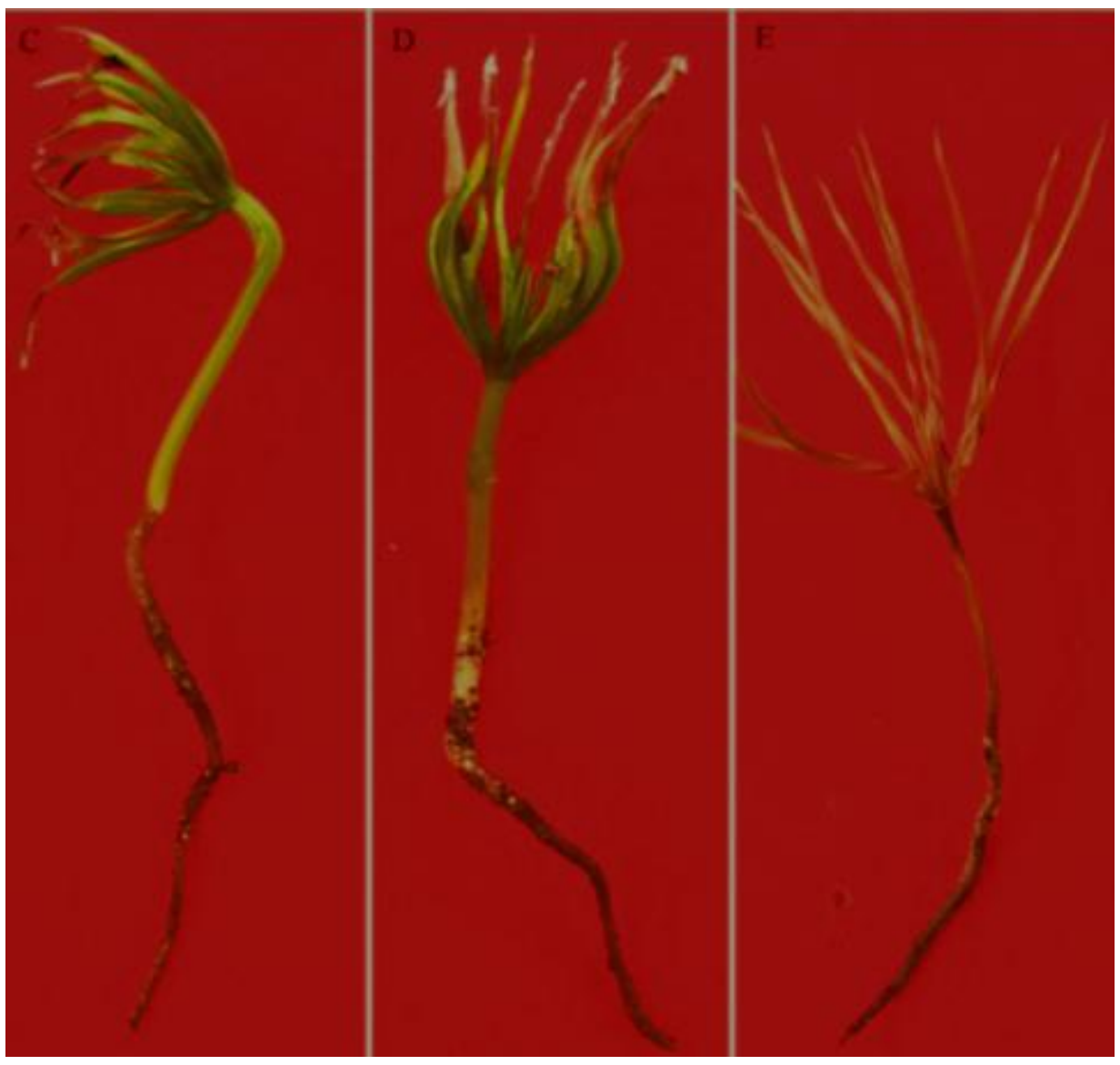

Fig. S1 Growth performance of *Pinus koraiensis* seedlings after infection by *Fusarium oxysporum* is characterized by leaf withering, root damage, and plant death.
